# Hamiltonian energy as an efficient approach to identify the significant key regulators in biological networks

**DOI:** 10.1371/journal.pone.0221463

**Published:** 2019-08-23

**Authors:** Shazia Haider, Kalaiarasan Ponnusamy, R. K. Brojen Singh, Anirban Chakraborti, Rameshwar N. K. Bamezai

**Affiliations:** 1 Department of Neurology, All India Institute of Medical Science (AIIMS), New Delhi, India; 2 School of Biotechnology, Jawaharlal Nehru University, New Delhi, India; 3 School of Computational and Integrative Sciences, Jawaharlal Nehru University, New Delhi, India; 4 Formerly at National Centre of Applied Human Genetics, School of Life Sciences, Jawaharlal Nehru University, New Delhi, India; INSERM U869, FRANCE

## Abstract

The topological characteristics of biological networks enable us to identify the key nodes in terms of modularity. However, due to a large size of the biological networks with many hubs and functional modules across intertwined layers within the network, it often becomes difficult to accomplish the task of identifying potential key regulators. We use for the first time a generalized formalism of Hamiltonian Energy (HE) with a recursive approach. The concept, when applied to the Apoptosis Regulatory Gene Network (ARGN), helped us identify 11 Motif hubs (MHs), which influenced the network up to motif levels. The approach adopted allowed to classify MHs into 5 significant motif hubs (S-MHs) and 6 non-significant motif hubs (NS-MHs). The significant motif hubs had a higher HE value and were considered as high-active key regulators; while the non-significant motif hubs had a relatively lower HE value and were considered as low-active key regulators, in network control mechanism. Further, we compared the results of the HE analyses with the topological characterization, after subjecting to the three conditions independently: (i) removing all MHs, (ii) removing only S-MHs, and (iii) removing only NS-MHs from the ARGN. This procedure allowed us to cross-validate the role of 5 S-MHs, *NFk-B1*, *BRCA1*, *CEBPB*, *AR*, and *POU2F1* as the potential key regulators. The changes in HE calculations further showed that the removal of 5 S-MHs could cause perturbation at all levels of the network, a feature not discernible by topological analysis alone.

## Introduction

Biological networks, embedded with fundamental information of the biological systems, are a part of the family of complex networks [1], which deal with the behavior of the components cross-talks and information processing [2], mechanisms of the governance of the systems and decision-making [3], design principles and architecture of the systems [4], and various other system-wide properties. Complex network theory is considered as one of the most useful mathematical techniques to understand the static [5] as well as dynamic [6] properties of complex biological systems [7]. In our previous work [8], we applied the tools of hierarchical networks and characterized the topological properties of a biological network associated with apoptosis, where the importance of hubs and constituting modules were reported. Hierarchical networks are of special interest because of their important structural properties (emergence of modules/communities and sparsely distributed hubs) [2, 9, 10] and self-organized working principle [11]. The studies of various parameters, e.g., probabilities of signal propagation, were able to provide mechanisms for establishing inherent properties of the system at various levels of the organization and identify hubs with significant biological roles. However, those topological parameters were unable to quantify the changes in the signal propagation as encountered by the hubs, when the network was subject to external or internal perturbations. Further, the topological analyses of the networks could not capture the amount of the perturbation. There have been methods proposed to study signal propagation, based on various techniques to identify disease genes with associated modules, which are applied to various disease-associated networks [12, 13]. Here, we adopted a different route, where we generalize the Constant Potts model (CPM) [14] and referred it as a method of HE.

In statistical physics literature, the Potts model [15] assumes a special Hamiltonian feature for a many-particle system on a lattice. The Hamiltonian is the sum of potential and kinetic energy at each lattice point of the system. The Potts model on a lattice structure was adopted to one on a network structure, by simply considering the positive links only in the configuration of well-defined communities [16]. It was generalized by considering internal links as positive and missing links as negative. The model was further simplified to the CPM and used for detecting communities in the complex networks [17]. The HE method based on this CPM was proposed with a further extension to system level organization in hierarchical networks [11]. The algorithm has already been applied to other networks involving breast cancer [17, 18] and turner syndrome networks [19]. It has been confirmed that the proposed HE method works very well and is sensitive to the identification of key regulators and corresponding pathways. There have been recent efforts in the analysis of networks focused on finding functional dependencies between the so-called hubs and their topological roles in the network [20]. However, hubs are not sufficient to control or regulate multi-functional or complex systems, such as any disease associated biological system. In such a system, modules are tightly interconnected and control the overall network organization by maintaining the network properties [21]. Each module has its own organization, cross-talking with its sub-modules at internal levels of the organization, which is minimally affected by neighboring sub-modules of other remaining modules. Considering the modules and hubs together along with the energy calculation could reveal potent regulators within any biological systems. Here, we applied HE method in the complex biological network system, which had the capability of distinguishing the significantly high-active and low-active regulators from the interacting nodes within the network. The key regulators that had higher HE value, were designated as significantly high-active, while the key regulators with a relatively lower HE value as compared to high-active regulators, were referred to as low-active key regulators. We propose that these high-active key regulators are significant motif hubs as compared to low-active key regulators because they play main and important role in network control mechanisms (as also known experimentally)[22]. Moreover, the HE approach helped us to quantity the perturbation caused by the removal of hubs. Thus, allowing us to filter out the most significant hubs out of a list of potential hubs within the network, which cause maximum perturbation. The HE approach, unlike Network topological Analysis (NTA) [23], turned out to be a simple and quick method to quantify the perturbation caused by the removal of hubs. The NTA, incidentally, had been proposed to be an important technique to understand topological properties of complex networks and their dynamics[20]. Most of the real networks fall within scale-free type [24], small world [25], random [26] and hierarchical [10] types. Hierarchical network is of special interest because of its important structural properties (emergence of modules/communities and sparsely distributed hubs) [2, 9, 10] and self-organized working principle [10, 11]. The emergence of modules/communities in this network type is of specific interest because they are believed to be corresponding to independent functions obeying their own laws [10] and their individual activities are nonlinear in nature [20]. The sparsely distributed hubs are supposed to interfere and control network stability [27] as well as other communities. Although network analyzer predicts the overall topology, the HE used in the present study provided not only global topology of the network but also obtained module-based topology at each level, which is the signature of the self-organization of the biological system. Among the hubs in the network, it is necessary to find the fundamental regulators, which perturb the network significantly. The fundamental regulators could influence the system even at modular level (network motif level). The motifs referred to here are by definition the 3-nodes or 4-nodes subgraphs, which cannot be further disintegrated [28]; and the motif level was defined as the level below which further breakup of the modules do not provide significant information of the network (cannot be achieved). The node that was present at all the levels of the systems, would significantly act as a fundamental regulator of the biological system, and could perturb the system drastically. Since the constructed network is a hierarchical modular, emergence of modules along with their functions is more prominent than the sparsely distributed hubs. Hence, removing of hubs do not cause breakdown of the network, which is seen in traditional scale-free networks. So, it does not obey “centrality-lethality rule” as in other scale-free networks, e.g. airport connections network. In our case, the hubs may significantly perturb the network “locally”, but at the global level the propagation of the signals may or may not be perturbed significantly. Therefore, developing the HE based method to recognize a potential key regulator in a biological system was crucial.

Systems Biology studies have a major impact on complex diseases, like cancer. The well-known biological process which in cancer is compromised effectively is apoptosis [29]. By using the experimental evidence involving a select set of apoptosis-regulatory genes (ARG) (S1 Table) [8, 30–35], we identified modules and sub-modules, which corresponded to independent functions, obeying the laws of modularity [10] and identified that their activities are non-linear in nature [20]. Therefore, quantifying their role as key ARG at the level of systems biology was essential, which we provide by using HE analysis of the biological networks.

## Methods

### Construction of apoptosis regulatory gene network

**Fig 1:**
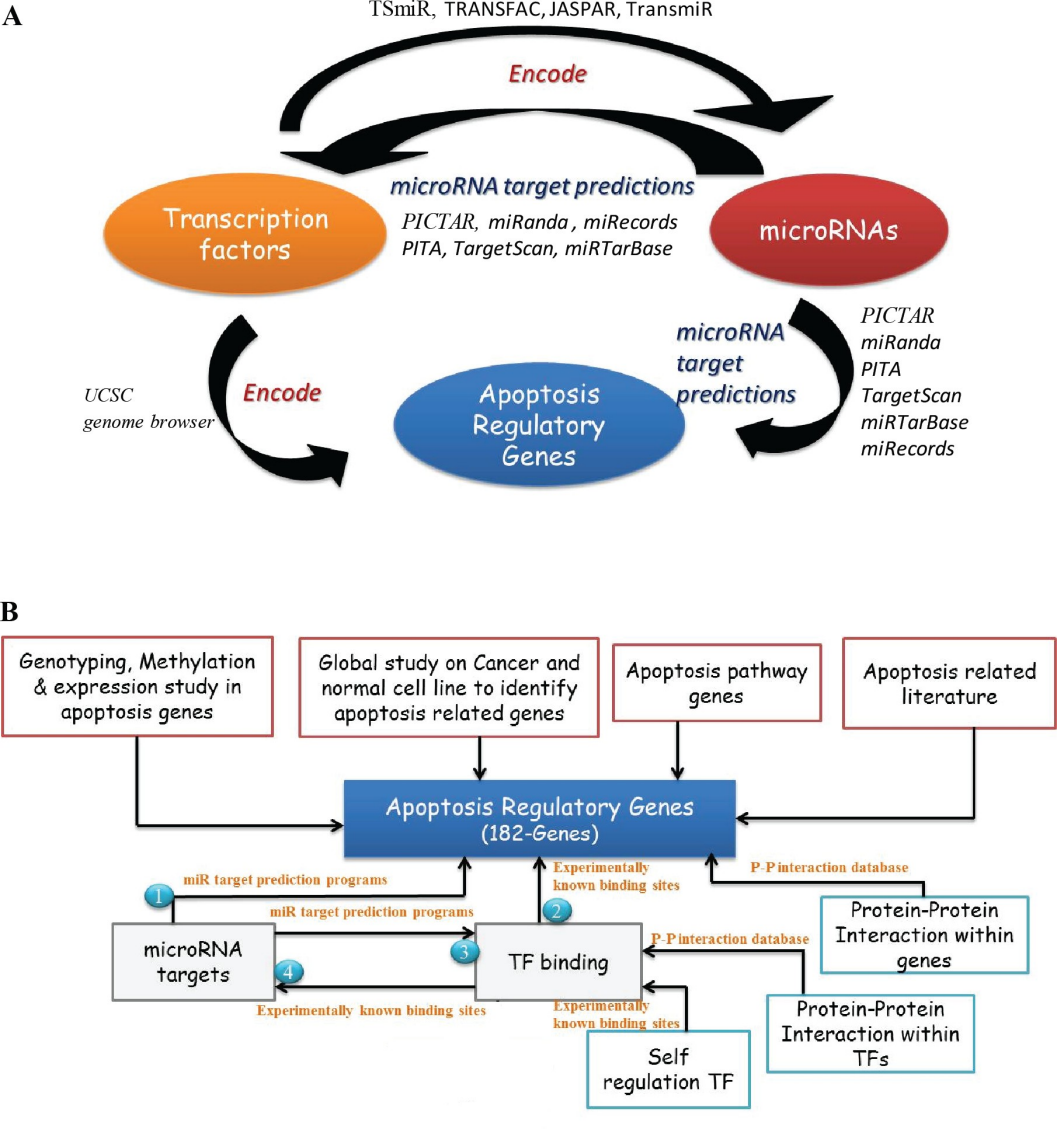
Regulatory relationship of apoptotic genes. **(A)** Relationship between ARGs, miRNA and TFs in the Apoptosis gene regulatory network. **(B)** Selection of apoptotic genes and their regulatory interactions.

The select set of apoptosis regulatory genes were based on previous experimental studies (S1 Table). In order to construct the AGRN, we subjected 182 ARGs for computational analysis and predicted the relationship between TFs (using three transcription factor binding databases: ENCODE [36], JASPAR [37] and TRANSFAC [38] and miRNAs (miRNA target prediction programs: PICTAR[39], miRanda[40], PITA[41] and TargetScan[42]. Experimentally validated miRNA targets for 182 genes from miRTarBase [43] and miRecords [44] databases were also retrieved (Fig 1A). Further, we also predicted the interacting partners of these 182 apoptosis regulatory proteins using various PPI (Protein-Protein Interaction) databases like DIP [45], IntAct [46], MINT [47], BioGRID [48], STRING [49] and HPRD [50]. Based on the relationships between TFs, miRNAs and Proteins we constructed and visualized network-using Cytoscape (V2.8.3) (S2 File) [51] (Fig 1B).

### Hub, modules, and sub-modules in the network

We applied the HE analysis to the network constructed on ARGN [8, 52]. Hubs and Modules in the network were identified using Network Analyzer [23] and MCODE (V1.32) [53], respectively. Further, modules were subject to MCODE for identification of sub-modules and sub-sub-modules. We considered all the modules, sub-modules and sub-sub-modules whose clustering coefficient values were less than or equal to unity (≤1). In the present study, all the modules, sub-modules and sub-sub-modules reflected as level-1, level-2, and level-3, respectively. These levels were used to compute the HE.

### Hamiltonian energy analyses

We considered a network given by a graph, *G* = *G* (*E*,*V*), with sets of constituting nodes *N* = {*k*},*k* = 1,2,…..,*N*, and edges *E* = {*e*_*ij*_},∀*i*,*j*∈*N*. The network was represented by the adjacency matrix *A*_*ij*_,∀*i*,*j*∈*N*, such that *A*_*ij*_ = 1 if i^th^ and j^th^ nodes are connected, otherwise zero. The Potts model [54, 55] was used to analyze properties of complex network [14]; and its simplified form, known as CPM or HE method, was used as a technique to detect communities in complex network [54]. If the network is hierarchical, which is constructed by systems level organization, then *G at level*−1 network is organized by *m* modules defined by graphs *g*_1_,*g*_2_,…..,*g*_*m*_, such that *g*_1_⊂*G*, *g*_2_⊂*G*,…,*g*_*m*_⊂*G* which was at *level-2*. Similarly, each module in *level-2* was organized by set of sub-modules at *level-3*, and so on.

The Potts model, which has successfully been applied to spin system in Statistical Mechanics, showed possibilities of the emergence of clusters of spins in the spin systems [56]. This concept of spin clusters reflected in the q-state Potts model was used by Reichardt and Bornholdt (RB model) to detect community structures in the complex network of spins by considering the properties of spin communities at ground state [57]. This allowed to estimate a resolution parameter σ of the emergence of communities in the network at various local minima. Then considering unweighted and undirected network, the Hamiltonian of a complex network [14, 54, 58, 59] is given by,

$$\begin{array}{c} H(c,\sigma )=-\sum_{i,j}(A_{i,j}-\sigma )\;\delta \;(c_{i.}c_{j})\\ \;=-\sum_{c}\left(E_{c}-\sigma N_{c}\right) \end{array}$$ (1)

where *c*_*i*_ and *c*_*j*_ are the communities of the network to which ith and jth nodes belong. A = [A_i,j_] is the adjacency matrix of the nodes in the complex network. If *H*^[1]^ is the Hamiltonian at *level-1*, then $H^{\left[1\right]}=-(E^{\left[1\right]}-\sigma N^{1})\;with\;E^{\left[1\right]}=\sum_{ij}A_{ij}^{\left[1\right]}\;and\;N^{\left[1\right]}=N\times N$. N^[1]^ is the total number of nodes in the level 1 of the networks. Similarly, for level-2 of the network, we have, $H^{\left[2\right]}=-\Sigma_{c_{1}=1}^{m_{c_{0}}^{\left[2\right]}}(E_{c_{1}}^{\left[2\right]}-\sigma N_{c_{1}}^{\left[2\right]})$ with $E^{\left[2\right]}=\Sigma_{c_{1}=1}^{m_{c_{0}}^{\left[2\right]}}E_{c_{1}}^{\left[2\right]},N^{\left[2\right]}=\Sigma_{c_{1}=1}^{m_{c_{0}}^{\left[2\right]}}N_{c_{1}}^{\left[2\right]}$ and $N_{c_{1}}^{\left[2\right]}=n_{c_{1}}\times n_{c_{1}}$, and so on, exhibiting formalism of systems level organization of complex hierarchical network. $m_{c_{0}}^{\left[2\right]}$ is the number of communities which belong to level number 2.

### Centrality-lethality rule of hub removal and its Hamiltonian energy

The centrality-lethality rule is widely believed to reflect the significance of network architecture in determining network function, a key notion of systems biology [60]. Hub node can also be present in a strongly interacting cluster of nodes (modules). HE based calculation were carried out for the network or modules by adopting two approaches. The first approach considered hubs, having interacting partners in the modules at different levels of the network and identified the modules where a particular hub has had maximum interaction at each level. Whereas, the second approach considered only the modules or sub-modules in which a particular hub was present. The hubs were removed in both the approaches to understand the significant changes in the HE of the system.

### Fundamental key regulatory hubs identification

**Fig 2:**
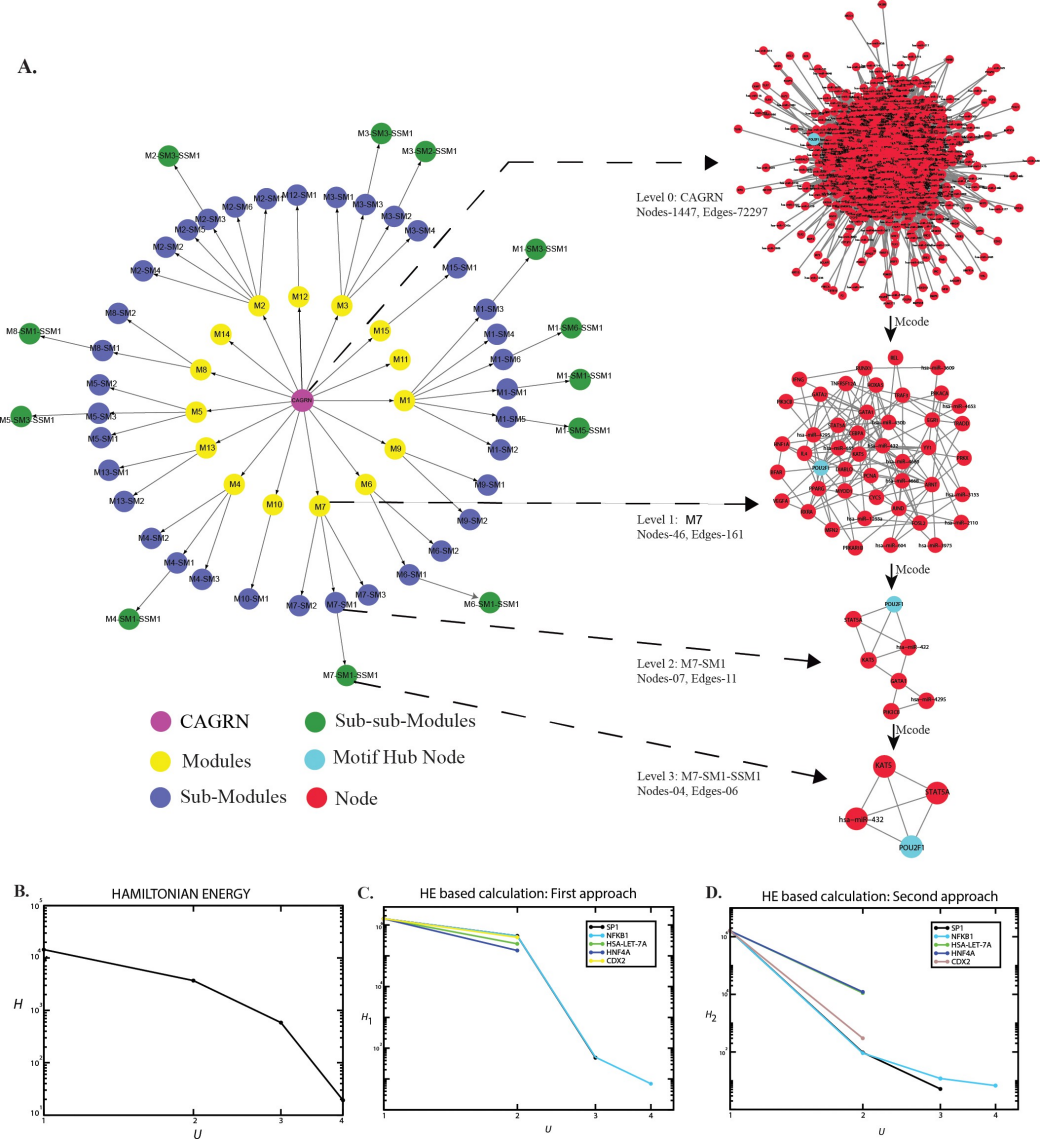
The Level in ARG network with Hamiltonian energy and probability of signal transduction of each hub. (A) The descendants of ARG network in the basic levels of the network, showing the significant existence of modules with their corresponding sub-modules and sub-sub-modules in the network. Zoom in of ARG network, modules with their corresponding sub-modules and sub-sub-modules in the network are indicated in dotted line. Hubs, which present up to the last level (motif level) of the apoptosis network, referred to as motif hub node, are shown in cyan color. (B) The Hamiltonian energy of the network calculated shows a decrease in its value as levels of the network (U) increases indicating faster information processing in the ARG network. (C) Hamiltonian energy, based on hub interacting partners in modules also show similar behaviour as in (B) indicating *NFk-B1* as a potential key regulator in the ARG network. (D) Hamiltonian energy based on the module having the particular hub indicating *NFk-B1* as a potential key regulator in the ARG network.

In the ARGN, the hub nodes which communicate with other nodes involved in various biological processes were identified using Network Analyzer [61]. Even though all the hubs are important regulators, only those hubs, which regulate the network from top to bottom (motif level), were considered as the most significant hubs. These hubs were termed as “fundamental” because they were deeply rooted in the network, served as the backbone of the network, and acted as basic information processors; known as signaling molecules throughout the network. Therefore, we ranked the top 100 hubs according to the number of degrees and identified the hubs, which presented up to the last level (motif level) of the apoptosis network. These identified hubs were referred to as MHs (Fig 2A) (Table 1) reconnoitering as potential key regulators in ARGN [17]. The MHs were subjected to HE calculation at different levels.

Level-0 (Complete Network): *F*_*x*_ = *K*_*x*_ /*H*0
Level-1 (Modules): *F*_*x*_ = *K*_*x*_ /*H*1
Level-2 (Sub-Modules): *F*_*x*_ = *K*_*x*_ /*H*2
Level-3 (Sub-Sub-Modules): *F*_*x*_ = *K*_*x*_ /*H*3

*F*_*x*_ = the ratio of degree of node x to the respective Hamiltonian energy

*K*_*x*_ = Degree of node

H(0–3) = 𝐻𝑎𝑚𝑖𝑙𝑡𝑜𝑛𝑖𝑎𝑛 𝑒𝑛𝑒𝑟𝑔𝑦 at the level of 0,1,2 and 3

**Table 1:** The 20 hubs with degrees (number of edges, or connections) at Level 0–3 are analysed. The above 11 identified hubs, which are present until the last level (motif level) of the apoptosis network, termed as MHs. While the remaining 09 hubs have very high degrees at Level 0, but subsequently their degrees are decreasing with the levels, with eventual absence at Level 3.

S.No.	Hubs	Degrees: L0	Degrees: L1	Degrees: L2	Degrees: L3
1	***GATA3***	276	24	7	6
2	***NFk-B1***	651	10	6	5
3	***POU2F1***	477	16	3	4
4	***CEBPB***	392	8	5	4
5	***AR***	390	9	4	4
6	***BRCA1***	276	9	4	3
7	***TFAP2A***	436	51	4	2
8	***ZEB1***	374	60	7	2
9	***hsa-miR-590***	294	31	2	2
10	***hsa-miR-20a***	288	31	3	2
11	***MEF2A***	318	43	2	2
12	***SP1***	714	15	4	0
13	***HNF4A***	711	89	23	0
14	***CDX2***	645	15	0	0
15	***MAX***	638	102	36	0
16	***JUND***	620	14	0	0
17	***TCF7L2***	620	114	0	0
18	***E2F1***	610	16	0	0
19	***EBF1***	587	103	0	0
20	***ETS1***	586	13	3	0

Based on the HE of 11 MHs, we categorized MHs into S-MHs and NS-MHs. The identification of significant/non-significant motif hubs was done in the following way. We took the maximum Hamiltonian energy of the MHs just before the motif level was taken as the threshold value of HE as a reference. A particular MH becomes significant if the HE calculated at the motif level is larger than this threshold Hamiltonian Energy (HE_T_) because the MH becomes significant and active till the last level of the network. Thus,

If HE_MH_ > HE_T_, then it is S-MH;
If HE_MH_ < HE_T_, then it is NS-MH;

Where, HE_MH_ = Hamiltonian Energy of Motif Hub, HE_T_ = Threshold Hamiltonian Energy.

Further, to validate the HE method, we constructed three different ARG-networks, which involved: (i) removal of S-MH nodes and their edges (S-MHs network), (ii) removal of NS-MHs nodes and their edges (NS-MHs network), and (iii) removal of both S-MHs and NS-MHs nodes and their edges (MHs network). These three different ARG-networks were subjected to module prediction, using MCODE. Each module at each level was subjected to NTA and HE calculation.

### Topological analyses of network and calculation of Δ HE

The statistical and functional significance of the ARGNs and HE based constructed network was calculated using Network Analyzer.

The probability of degree distribution, *P*(*k*), of a network is defined as the probability a particular node has degrees between ˈkˈ and ‘k+dk,' which can be used to calculate for example, how many degrees on an average a node may have. Another definition of *P*(*k*) in discrete network is the ratio of the number of nodes having k degree (n_K_) to the total number of nodes in the network, $P_{K}=\frac{n_{K}}{N}$. It can be used to capture the network structure, identification of hubs and modular organization of the network [62]. The network constructed was found to obey power-law degree distribution *P*(*k*)~*k*^−*γ*^, indicating the scale-free nature of the network [24, 27] where γ is a parameter that can identify the different topological structures of a scale-free network: (a) if γ is 2≤*γ*≤3, few large hubs hold a number of smaller hubs and large number of individual nodes together [24], (b) if *γ*≤2, inherent modular structure in the network emerge, known as hierarchical network where the role of modules is more important [10], and (c) if *γ*>3, the hubs are irrelevant, losing various scale-free features in the network, qualifying the network as random network [11].

Clustering coefficient of a network characterize how strongly node(s) neighborhood(s) are connected internally. It is defined as the ratio of the number of triangular motifs a node has with its nearest neighbor to the maximum possible number of such motifs. For an undirected network, clustering co-efficient of ith node can be obtained by $C{(K}_{i})=\frac{{2E}_{i}}{K_{i}(K_{i}-1)}$, where, *E*_*i*_ is the number of connected pairs of nearest neighbor of *i*_*th*_ node, and *K*_*i*_ is the degree of the respective node. For scale free networks *C(K) ~* constant, whereas, for hierarchical network it follows a power law, *C(k) ~ k*^*−α*^, with α ~1 [10, 11, 25, 63]. The average clustering coefficient ⟨*C*(*k*)⟩ identifies the overall organization of clusters formation in the network. Similar to P(k), ⟨*C*(*k*)⟩ may depend on network size [24] and characterizes various properties of the network: (i) for scale free and random networks where ⟨*C*(*k*)⟩ is independent of k ⟨*C*(*k*)~*constant*⟩, [11] and (ii) for hierarchical networks where ⟨*C*(*k*)⟩ follows power law scaling behavior, *C*(*k*)~*k*^−*β*^ with β ~ 1 [10].

The neighborhood connectivity of a node is the number of connected neighbors with it and characterizes the correlation pattern of connectivity of interacting nodes in the network [27]. This connectivity correlation measured by defining a conditional probability *P*(*k*′_*n*_|*k*_*n*_) which is the probability of making a link from a node having degree k_n_ to another node of degree k_n_ [64]. Then the average neighborhood connectivity of nodes with connectivity k_n_ is given by, $C_{n}(k_{n})=\sum_{{k\prime }_{n}}{k^{\prime }}_{n}P({k^{\prime }}_{n}|k_{n})\sim k_{n}^{-\propto }$, [65] following a power law scaling behavior with α <1 for most of the real networks [27, 64]. If C_n_(k_n_) is an increasing function of k_n_ (for negative values of α) then the topology of the network show assortative mixing [65] where high degree (the number of edges per node) nodes have the affinity to connect to other high degree nodes in the network. However, *C*_*n*_(*k*_*n*_)~*k*_*n*_^−*α*^ with positive values of α, is the signature of the network having hierarchical structure [64], where low degree nodes tend to connect high degree hubs [65], and the few high degree hubs present in the network try to control the low degree nodes.

The statistical analysis for these networks was carried out through two methods:

**Method 1:** By removing the MHs nodes and their edges from the ARGN at all levels. The removing of MHs carried out in three different ways: removing (i) S-MHs, (ii) NS-MHs and (iii) both S-MHs and NS-MHs. Each module thus was subjected thrice for network analyses like calculating in-degree and out-degree distribution, all-neighborhood connectivity, in-neighborhood connectivity, out-neighborhood connectivity and average clustering coefficient.

**Method 2:** After the removal of (i) S-MHs nodes and their edges, (ii) NS-MHs nodes and their edges and (iii) both S-MHs and NS-MHs nodes and their edges, the networks were reconstructed. These three networks were subject to module prediction, using MCODE and predicted modules at each level analyzed using a Network Analyzer. The average values were calculated for, in-degree and out-degree distribution, all-neighborhood connectivity, in-neighborhood connectivity, out-neighborhood connectivity and average clustering coefficient of each module, sub-modules, and sub-sub-modules of the three networks at different levels.

### HE calculation of fundamental hub removed apoptosis network

The HE of network/modules/sub-modules/sub-sub-modules at each level after removing fundamental regulators from the network was calculated, based on the steps mentioned above in section “Hamiltonian energy analysis.” To calculate the perturbation in the fundamental hub removed apoptosis network, we compared its HE with the HE of ARGs Network. The difference in the HE of the ARGs network and the fundamental hub removed apoptosis network measures the perturbation caused by the fundamental regulators.

$${\Delta HE}^{L0}={HE}^{L0}-{HE}_{\beta }^{L0}$$

$${\Delta HE}^{L1}={HE}^{L1}-{HE}_{\beta }^{L1}$$

$${\Delta HE}^{L2}={HE}^{L2}-{HE}_{\beta }^{L2}$$

$${\Delta HE}^{L3}={HE}^{L3}-{HE}_{\beta }^{L3}$$

Here,

L = Levels in the network; β = Fundamental hub removed network

## Results

### Hamiltonian energy of hubs

Complex natural networks are self-organized [66, 67] and have various levels of organization, which have a self-similar constitution at each level of organization [68], down to the fundamental level where basic organizational units are motifs [69]. The complete network (level-0) constructed by the interaction of communities at level-1; the sub-communities of all communities, building the level-1 network at level-2, and so on. If ∪ levels of organization organize the network, the properties of the complex network are due to coordinated behaviors of the networks at various levels. Hence, the Hamiltonian of the complete network *H*^[1]^ can be derived from the Hamiltonians at the lower levels,level−1,level 2,…,level−∪. Proceeding in the same way as in equation (Eq 1, described in the methods), we have,

$$\begin{array}{l} H^{\left[\cup \right]}=-\sum_{c_{1}=1}^{m_{c_{0}}^{\left[2\right]}}\;\sum_{c_{2}=1}^{m_{c_{1}}^{\left[3\right]}}\;\sum_{c_{3}=1}^{m_{c_{2}}^{\left[4\right]}}\ldots .\sum_{c_{\cup -1}=1}^{m_{c_{\cup -2}}^{\left[\cup \right]}}H_{c_{1}c_{2}\ldots .c_{\cup -1}}^{\left[\cup \right]}\\ \;H_{c_{1}c_{1}\ldots .c_{\cup -1}}^{\left[\cup \right]}=E_{c_{1}c_{2}\ldots .c_{\cup -1}}^{\left[\cup \right]}-{\sigma N}_{c_{1}c_{2}\ldots .c_{\cup -1}}^{\left[\cup \right]} \end{array}$$ (2)

Here, $m_{c_{0}}^{\left[2\right]},m_{c_{1}}^{\left[3\right]},\ldots .,m_{c_{\cup -2}}^{\left[\cup \right]}$ are numbers of communities/sub-communities/sub-sub-communities…at level−1,level 2,….,level−∪ respectively. The negative $H_{c_{1}c_{2}\ldots ..c_{\cup -1}}^{\left[\cup \right]}$ gives Hamiltonian function of *c*_∪−1_*th* community of level−∪ constructed from *c*_∪−2_*th* community at level−(∪−1)… constructed from the *c*_1_*th* community. Thus, from Fig 2A, we have for our ARGs network,

### Hamiltonian energy

$$H^{\left[4\right]}=-\sum_{c_{1}=1}^{m_{c_{0}}^{\left[2\right]}=15}\;\sum_{c_{2}=1}^{m_{c_{1}}^{\left[3\right]}=36}\;\sum_{c_{3}=1}^{m_{c_{2}}^{\left[4\right]}=12}H_{c_{1}c_{2}c_{3}}^{\left[4\right]}$$

$$H_{c_{1}c_{2}c_{3}}^{\left[4\right]}=E_{c_{1}c_{2}c_{3}}^{\left[4\right]}-{\sigma N}_{c_{1}c_{2}c_{3}}^{\left[4\right]}$$

wheretheconstant,σ=0.8(theresultsarerobustw.r.t.thisparametervalue)

The HE calculation for a network within the formalism of CPM considers contributions from the organization of nodes and edges in a competitive manner, and this energy is used in organizing or re-organizing the network at various levels. This approach can also magnify the significant changes in the network organization when it goes down to various levels of organization, which capture the importance of hubs in the network as well as at the modular level. Therefore, HE formalism proves to be a useful technique for considering variations in the network organization.

The HE was calculated for hubs at each level of all possible modules in the network (Fig 2A). The HE of the ARGs network are plotted as a function of network levels, U (Fig 2B, 2C and 2D). We found that the energy distribution in the primary network is highest and starts decreasing as the level of organization increases (Fig 2B). Since HE is dependent on the competition between nodes and the edges for a fixed resolution parameter value (gamma symbol), the decrease in HE indicates the dominance of the interacting edges over the network size, indicating fast information processing. Similarly the behaviour of the HE calculated using first approach and second approach (as discussed above in methods) (Fig 2C and 2D) were found to be similar nature as in Fig 2B. From the Fig 2C and 2D, it is found that *NFk-B1* participated till the last level, which indicated the importance of this key regulator. We found that the regulators were tightly interconnected and could pass signals quickly. The nodes in a tight cluster generally have large number of edges (high degree nodes). Since strength of information propagation in the network depends on the number of edges, the tight cluster could propagate/receive faster information; for example, triangular motifs considered to be controlling components of a network [8]. The calculated HE of *NFk-B1* (First approach: by considering the hub and its highly interacting modules at each level) showed a sudden decrease in its value after level-2 and maintained stable behaviour (Fig 2C). The same was true when hubs HE calculated only with its associated modules (Second approach) (Fig 2D). Thus, indicating that *NFk-B1* is a potential key molecule regulating the apoptosis network system [52]. The hubs that present from primary to the last level of the network are potential key regulators of the network. The S1 File includes the list of all the hubs that are not potential key regulators. We propose to perform a clinical trial on the predicted key regulators for potential drug targets. It proved that not all hubs could regulate the network; and the hub that is present until the last level may have the capability to regulate the network. Our results suggest it is necessary to identify the hubs present until the motif level, known as MHs.

### Identification of motif hubs and their HE analysis

**Fig 3:**
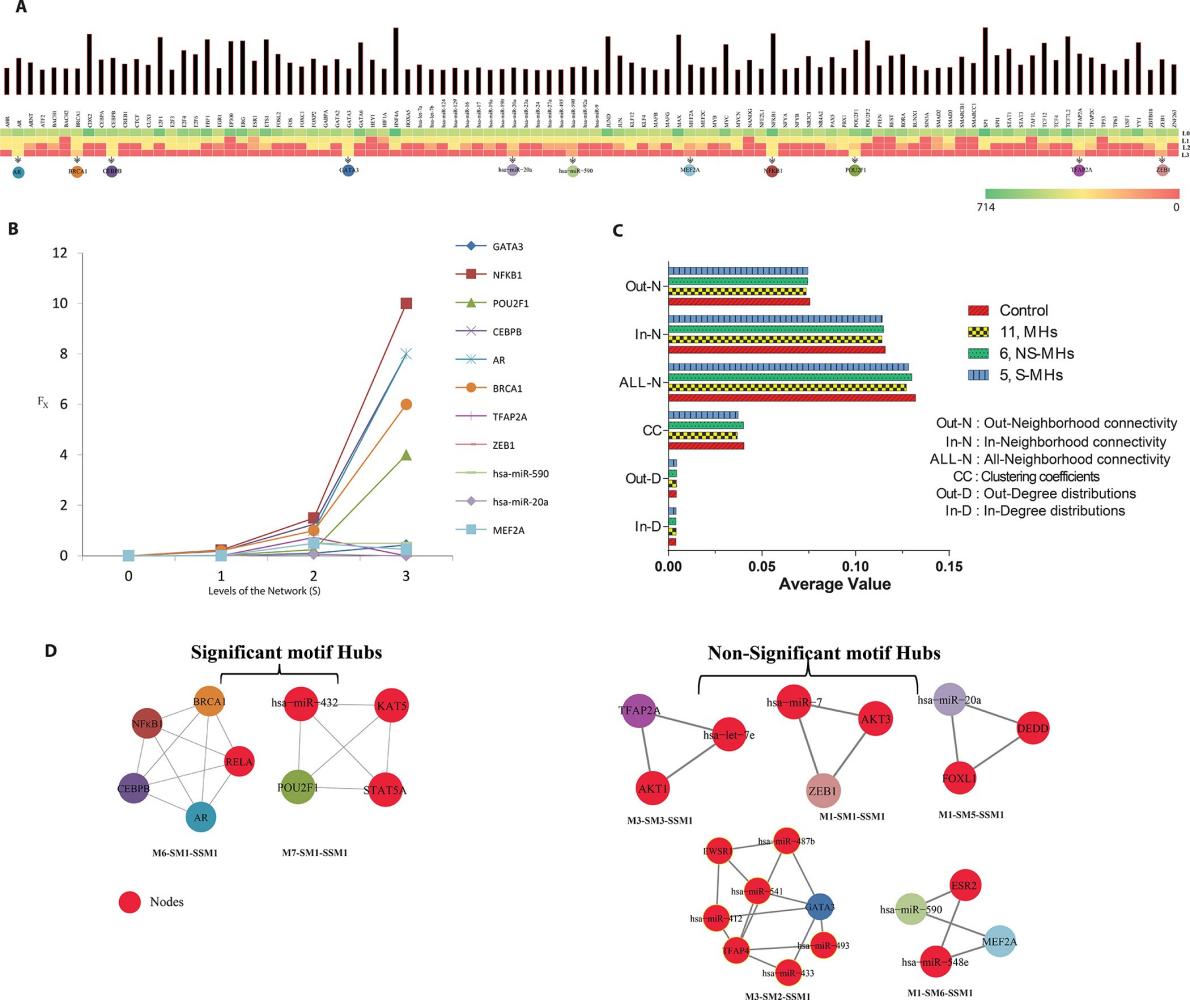
Analysis of the different levels in the ARG network. **(A)** The degree of top 100 hubs at each level of the ARG network (see S3 File for details). The color bar represents the strength of the degree at each level. **(B)** The Hamiltonian energy of the motif hubs at each level of the network. Each motif hub is highlighted with different color code. **(C)** Statistical topological properties in comparison to the complete and motif hub removed network. **(D)** The 5 significant motif hubs and 6 non-significant motif hubs are present in 7 sub-sub-modules of the ARG network. They are represented with the same color scheme as in (B).

From the ARGs network, we found 15 modules, which further divided into sub-modules, and sub-sub-modules up to 3^rd^ levels. The topological structure of the network manifested the presence of various functional modules or sub-networks and its organization [10]. All hubs may be important, but it is essential to understand their significance. The hub nodes, which regulate the network from the top (complete network) to bottom level (motif level), are the fundamental regulators. To analyze this in the present study, among the100 hubs with highest degrees (number of edges, or connections) (S3 File) we identified 11 hubs, which are present until the last level (motif level) of the apoptosis network (Table 1) (Fig 3A). These 11 fundamental regulators (*GATA3*, *NFk-B1*, *POU2F1*, *CEBPB*, *AR*, *BRCA1*, *TFAP2A*, *ZEB1*, *hsa-miR-590*, *hsa-miR-20a*, and *MEF2A*) were termed as MHs. It is not necessary that all MHs should regulate the network positively. The NTA could not differentiate the high-active and low-active regulator in the filtered hubs. Even though we could apply permutation and combination of centrality-lethality rule of hub removal to provide a nonspecific classification of the regulators, but it is computationally complex or laborious. The **“c**entrality-lethality rule” generally observed in scale-free networks, where removing hub/hubs cause network breakdown. In our case, the ARG network followed a hierarchical structure, where functions of the emerged modules were more significant than those of the hubs do. Hence, the network does not collapse on the removal of hub/hubs. A plausible reason could be self-organization property of the network, where a central control system is not present. We have done “hub knockout” experiment, where we found changes in the topological properties of the network, but we did not get network collapse upon removal of the hub/hubs [52]. Therefore, HE was calculated to distinguish the high-active and low-active regulators (Fig 3B) based on their HE values. The key regulators were high-active, which had high HE value, and were considered as significantly high-active. The key regulators having significantly low HE values as compared to high-active regulators were turned as low-key regulators. We claim that these high-active key regulators are significant motif hubs as compared to low-active key regulators because they play main and important role in the network control mechanisms. HE proved to be a simpler and easier way to quantify and classify the MHs than topological analysis. Out of these 11 MHs, we identified only 5 S-MHs (*NFk-B1*, *CEBPB*, *AR*, *BRCA1* and *POU2F1*) having significantly high HE score; whereas 6 NS-MHs (*GATA3*, *MEF2A*, *TFAP2A*, *ZEB1*, *hsa-miR-590* and *hsa-miR-20a*) showed very low HE score. Out of the 5 S-MHs (high-active regulators) predicted by HE method, *NFk-B1* was found to be a potential key regulator and we propose to verify experimentally the rest of the key regulators.

### Critical comparison of HE approaches and NTA

We constructed four networks (ARGs, MHs, S-MHs and NS-MHs) which further were subjected to network topological and HE analysis. The network topological properties like, in-degree, out-degree, clustering coefficient, All-neighborhood, In-neighborhood, and Out-neighborhood were calculated for each network. In level-0, both the HE analysis and NTA failed to show the effect of key regulators significantly, which could be achieved in our HE method by incorporating signal propagation method proposed in Nature Physics [20]. In previous works [8], we had found using this method that the signal propagated up to a maximum of level 3 (threshold could be 3) beyond which the propagation reduced as the level increased. In NTA, two parameters, namely, the clustering coefficient and all-neighborhood connectivity, showed insignificant marginal perturbations in the network (Fig 3C). In case of HE, since we considered different levels of the network (which were not considered in NTA) and computed the HE values, we could find out the significant key regulators (Fig 3B).

**Fig 4:**
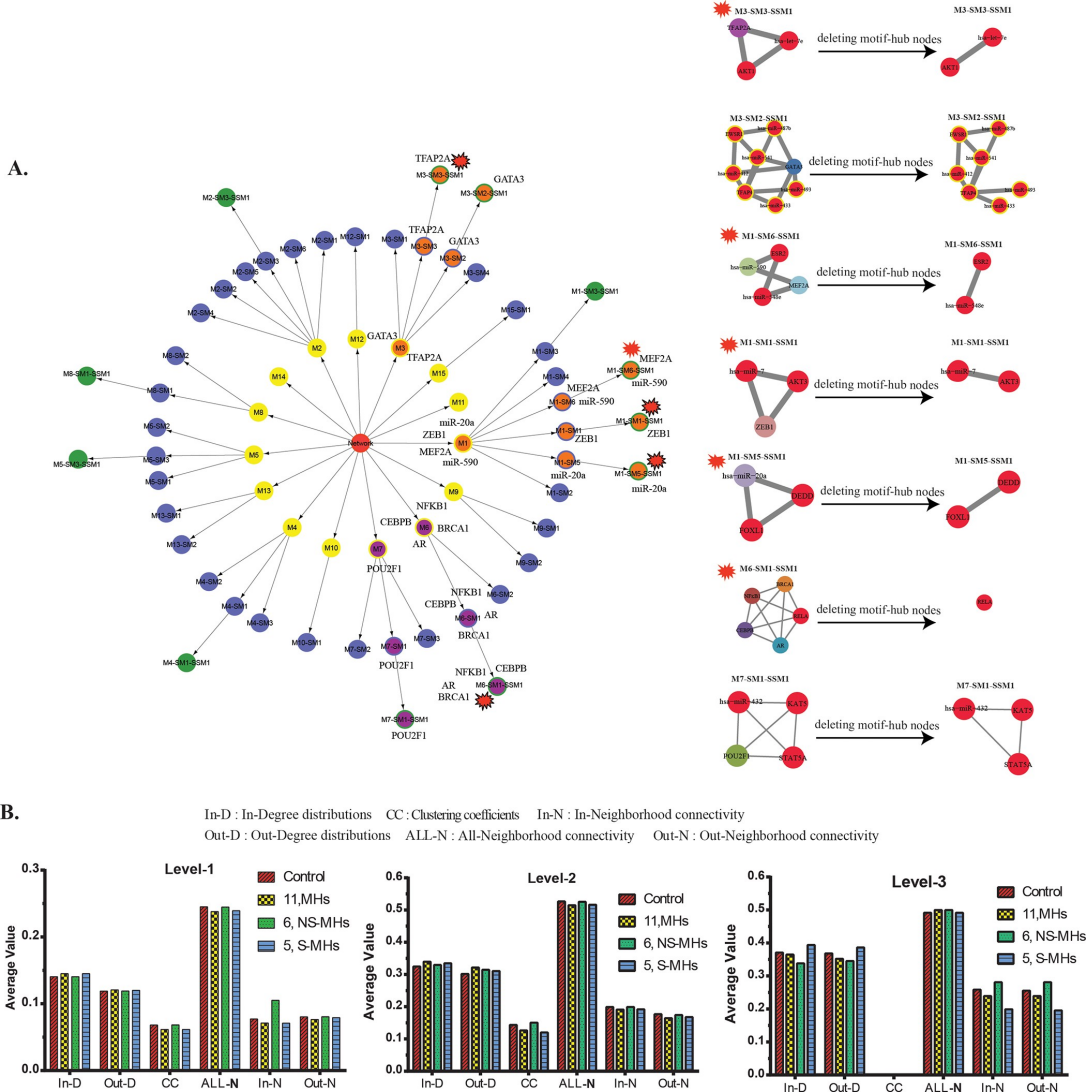
Influence of significant and non-significant motif hubs in the network. **(A)** Network representation of each level and effect of 6-MHs (nodes filled with orange) and 5-MHs (nodes filled with purple). The graphical representation showed loss of levels after removal of MHs from the network. Illustration to show what happens to the sub-sub-modules when the MHs are deleted/knocked-off; out of the 7 sub-sub-modules, 5 of them are severely perturbed (modular structure breaks down), but the remaining 2 have partly intact modular structure. **(B)** Network topological analysis on complete, motif hubs, significant and non-significant motif hubs. The analysis was carried out at all the three levels.

The HE and network topological analysis were carried out in two different ways as explained in method section (method-1 & 2) to quantify the perturbation at each level of the networks. Using method-1, out of 5 S-MHs, 4 MHs (*NFk-B1*, *BRCA1*, *CEBPB*, *and AR*) clustered in module-6 and 1MHs (*POU2F1*) present in module-7. In the case of 6 NS-MHs, 4 MHs (*miR-20a*, *ZEB1*, *MEF2A*, and *miR-590*) appeared in module-1 and 2 MHs (*GATA3* and *TFAP2A*) in module-3 (Fig 4A). Removal of NS-MHs like *TFAP2A*, *ZEB1*, and *miR20a* resulted in the nonexistence of three modules at the 3^rd^ level. Removal of four S-MHs from module-6 leads to the nonexistence of a module at 2^nd^ level itself which also affected the 3^rd^ level. This analysis showed that removal of S-MHs could disturb the network in the very first (few) levels, compared to NS-MHs. Thus, it proved that HE method could effectively distinguish the high-active/low-active regulator, or significant/non-significant regulators through HE values. In NTA, we found that the S-MHs network showed a slight perturbation only at the third level with an increase in in-degree and out-degree compared to other three networks (Fig 4B). It also showed a decrease in in-neighborhood and out neighborhood; whereas NS-MHs network showed the visa-versa result.

**Fig 5:**
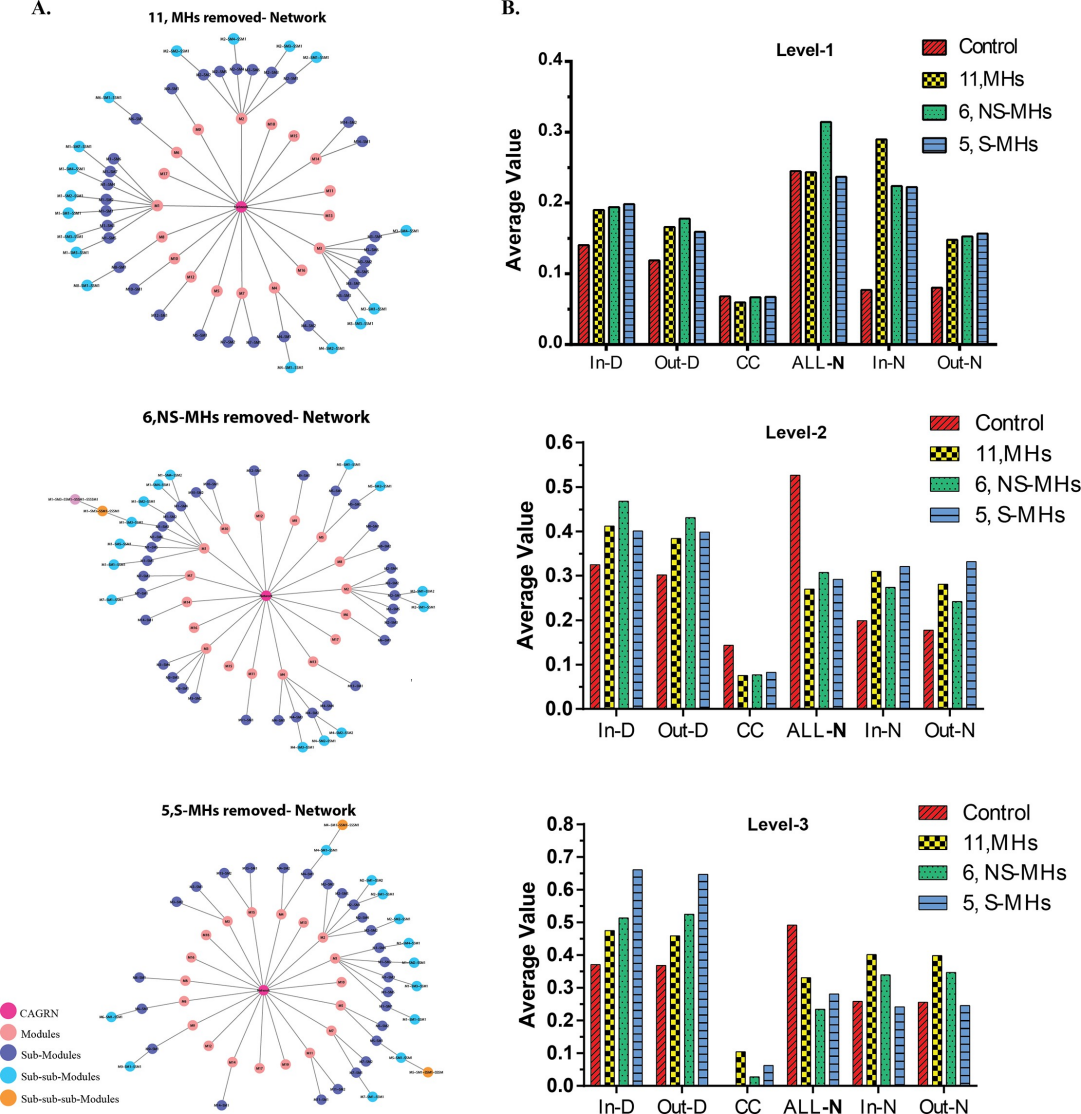
Reconstruction of modules and levels based on motif hubs. **(A)** Network representation of modules and levels based on the removal of 11-MHs, 6-MHs, and 5-MHs. Each level is represented in different color and node represent each module. **(B)** Network topological analysis on reconstructed modules and levels based on the removal of 11-MHs, 6-MHs, and 5-MHs. The comparison of complete, motif hubs, significant and non-significant motif hubs networks. The analysis is carried out at all the three levels.

Through HE calculation, we found S-MHs perturbed the network effectively. Although method-1 suggested the importance of S-MHs, the network used for the analysis was not able to reconstruct after removing the motif-hubs and their edges. Therefore, to understand which of the 11 MHs is most important, we reorganized and reconstructed the network using method-2 (described in the methods section), by removing these MHs (11 MHs, 5 S-MHs, and 6 NS-MHs) and further subjected to module prediction using MCODE (Fig 5A). The NTA showed significant differences in the network properties between control and reconstructed networks with higher values in most of the network topological parameters namely (in-degree, out-degree, clustering coefficient, All-neighborhood, In-neighborhood, and Out-neighborhood) at all levels (Fig 5B). However, the NTA failed to distinguish the significant/non-significant regulators. In the case of neighborhood connectivity, the removal of MHs, S-MHs, and NS-MHs disturbed the networks effectively at level-2 and 3.

The network topological analysis depicted that MHs were important for network perturbation, but it was not possible to quantify. NTA when applied in a recursive or non-recursive manner, it could only identify the hub in the network and its role; whereas HE could identify the key regulator in any biological network, in a recursive, easy and quick manner (Table 2) because of the fact that the network analysis was carried out within the framework of Potts model and formulation, and the network analysis algorithms have low orders of complexity.

**Table 2:** Comparison of Hamiltonian energy and network topological analysis calculation.

S.No.	Hamiltonian Energy	Network Topological Analysis
1.	It gives both qualitative and quantitative approach to understanding the perturbation in a network.	It gives a qualitative approach to understand the perturbation [8, 52, 54].
3.	It is a recursive way of identification of hubs. It is simpler, straightforward and meaningful.	This approach is recursive as well as non-recursive [8, 52, 54].
4.	The hubs can classify as significant and non-significant regulators.	Cannot be used to classify the significant and non-significant regulators [8, 52, 54].
5.	A single value of the HE helps in finding the key regulators in the network.	Several parameters/ characteristics (degree distribution, neighborhood connectivity, Centrality, and clustering) should be computed and analyzed to find the important regulators [8, 52, 54].
6.	No ambiguity or arbitrariness as a single parameter value of HE differentiates the importance of regulators.	Hard to rank the relative importance of the regulators as several parameters/characteristics may turn out to be difficult to compare [8, 52, 54].
7.	Less cumbersome to find key regulators; it can be made easy at first step rather than going to a different combination of hub removal.	It is a cumbersome method to find the key regulator which also requires permutation combination of hub removal [8, 52, 54].
8.	Reduces the complexity of filtering key regulators. It reduces the number of hubs and facilitates a quick analysis of the importance of significant hubs.	It is very cumbersome computationally to compare and find the key regulators [8, 52, 54].
9.	HE analysis splits the network into its subsequent sub-networks and calculates energy.	Network topological analysis considers the complete network in all the parameter/characteristics calculation [8, 52, 54].
10.	The identified key regulators through HE can be validated using ΔHE calculation, which is quick and effective.	Validation of key regulators identified using Network properties/characteristics needs a different combination of hub removal and calculation of several parameters, which can be laborious and elaborate [8, 52, 54].

In classical mechanics, the Hamiltonian gives the total energy of the system (under certain constraints).

### Hamiltonian energy calculation of motif hubs networks

**Fig 6:**
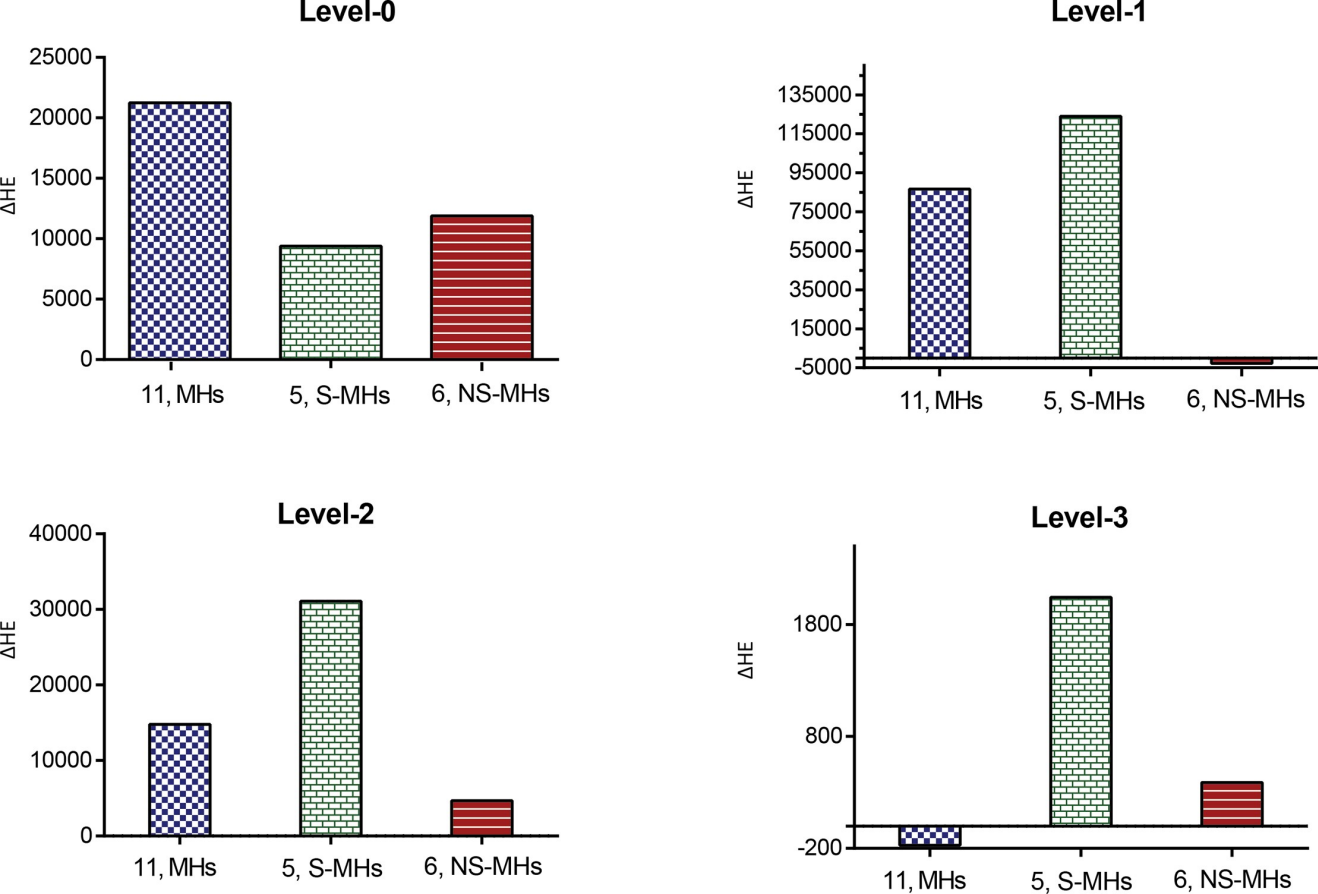
Delta Hamiltonian energy calculation of motif hub networks. The difference in Hamiltonian energy calculated for each levels of the three different networks (MHs, S-MHs & NS-MHs), along with control network.

The HE was calculated for each level in the complete network (control/ARGN), MHs network, S-MHs network and NS-MHs network. The delta HE calculated at each level, which is the difference in energy compared to the control network/AGRN (Fig 6). At level-0, MHs network showed a slightly higher perturbation compared to S-MHs and NS-MHs network. In the Level-1, 2 and 3 S-MHs network showed higher perturbation as compared to other networks. This analysis proved that S-MHs were the fundamental key regulators; in comparison to the NS-MHs network, where the removal of six hubs did not significantly affect the network. HE calculation helped us in deducing a small number of hubs, which played a critical role in the complex network. Thus, the HE method could identify potential key regulators within high degree hubs in a biological network; providing a novel approach to quantify the significant (high-active) and non-significant (low-active) regulators within a biological network. We have also done hub removal experiment in order to understand the sensitivity of the perturbing the network and their organization (Fig 6). We showed that the calculated HE gets changed significantly when the few hubs are removed that is the network is perturb indicating sensitivity of the network to the perturbation. However, the network does not breakdown and retains its organization keeping hierarchical features. This indicates that the network is sensitivity to the perturbation but try to retains its network organization and the properties, which is pretty robusts property.

## Discussion

Hamiltonian energy in a complex network is a measure of the total energy in the system, and as the network structure changes, the value of the HE also changes. In this paper, the HE was used to compute for different distributions and organizations of edges (wiring/rewiring of edges) with specified weights and directions among the constituting nodes in the network. The sub-modules and sub-sub-modules in a network have their organization at different levels [10, 63]. HE based analysis provided an assessment tool to learn about the behavior of the network in the context of the modules. Thus, the self-organization of the system was based on the energy distribution, suggesting to study the HE [70] of the network at different levels.

In one of our previous study [8, 52], we identified hubs in the ARGs based on topological properties; but could not measure the perturbation caused by them in the network. The removal of main hub *NFk-B1* or its combination failed to provide the qualitative and quantitative information on perturbation, using topological analysis. In order to understand the perturbation and find out the importance of hub qualitatively and quantitatively, we adopted HE method. Both modules and degrees were considered in the HE analysis, which involved less computation. In NTA, the removal of hubs is carried out at the complete network or level-0 and any perturbation caused due to this removal of one or more hubs is not discernable. Whereas, in HE the hubs present up-to the last level allowed a recursive approach to reduce the number of hubs and facilitate quick analysis, as well as attach importance to the significant hubs. In all filtered hubs, there are limitations within topological properties to differentiate the significant (high-active) and non-significant (low-active) regulators. The HE approach proved to be a recursive way to identify fundamental key regulators of the network after reducing the number of hubs; and finally establish an important one. We applied HE method for the first time in a biological system and found it was better than topological analysis to identify self-organization of the system. Additionally, this approach helps validate the possibility of self-organization of the system derived from the topological analysis. Network analyzer provides information about nodes and edges, degree distribution properties that follow a power-law distribution, suggesting that the network is stable and self-organized with the limitation of inclusion of both nodes and edges. However, in the HE approach, we noticed that such a redundancy is taken care of, using modules, which were strongly interconnected. Network analyzer property provided the information of key regulators (hubs) in a network. Whereas HE approach clearly stated the importance with respect to the stability and regulation of self-organization of the network, viz. a viz., the key regulatory molecules. In our study, we have demonstrated the utility of both these approaches in establishing the role of key regulators in the stable and self-organized network. The method of HE provides a novel approach not only to validate the assumed self-organization of a network through the network analyzer analysis; but also to provide a better understanding of the qualitative and quantitative perturbation in the network. Following Kauffmann definition of self-organization in which fractality in the system and absence of central control mechanism are the ingredients to establish self-organization in a system [67, 69]. The power law behaviour in the network is the signature of fractal nature in the network, which is the indicator of self-organized behaviour. Further, the network follows hierarchical properties there is no central control system where removal of hubs do not cause network breakdown. Gene network is self-organized in the sense that the network tries to maintain the two properties mentioned above despite any perturbation is given to the network.

In summary, we have used for the first time a generalized formalism of HE with a recursive approach for the identification of potential regulators in a biological network. The concept, when applied to ARGN, it helped us to identify 11 Motif hubs (MHs), which influenced the network until motif levels. The approach further classified them into 5 significant motif hubs (S-MHs) and 6 non-significant motif hubs (NS-MHs); where the significant motif hubs had high HE values and were considered as high-active key regulators in network control mechanism; while the non-significant motif hubs had relatively low HE values and were considered as the low-active key regulators in network control mechanism. Further, we compared the results of the HE analyses with respect to the topological characterization, for three conditions after: (i) removing all MHs, (ii) removing only S-MHs, and (iii) removing only NS-MHs from the ARGN, independently. This procedure allowed us to cross-validate the presence of 5 S-MHs, *NFk-B1*, *BRCA1*, *CEBPB*, *AR*, and *POU2F1* as potential key regulators. We found that *NFk-B1* was the most significant among the five. The changes in HE calculations showed that the removal of 5 S-MHs could perturb the network at all levels of the network, which was not discernible by topological analysis alone. In this manner, HE may be useful in identifying the significant fundamental key molecule in any other biological network as well.

## Supporting information

S1 Table A selection of apoptosis-regulatory genes (ARG). The select set of 182 apoptosis regulatory genes were based on previous experimental studies in order to construct the AGRN.
(DOCX)

S1 File Top 100 hubs and MHs with their respective degrees. It includes the list of all the hubs that are not potential key regulators. The hub that is present until the last level (motif level), known as MHs may have the capability to regulate the network.
(XLSX)

S2 File Construction of apoptosis regulatory gene network. Interacting partners of 182 ARGs based on the relationships between TFs, miRNAs and Proteins.
(XLSX)

S3 File Identification of motif hubs. Among the100 hubs with highest degrees (number of edges, or connections) we identified 11 hubs, which are present until the last level (motif level) of the apoptosis network.
(XLSX)
