# Supplementary material for: Hamiltonian energy as an efficient approach to identify the significant key regulators in biological networks
Source: PLoS One. 2019 Aug 23;14(8):e0221463. doi: 10.1371/journal.pone.0221463 (PMC6707611; doi:10.1371/journal.pone.0221463)
Supplement: S1 Table — The select set of 182 apoptosis regulatory genes were based on previous experimental studies in order to construct the AGRN. (DOCX) [file pone.0221463.s001.docx]

| **S.No** | **Gene Name** | **Gene-ID** | **Uniprot-ID** | **Description** | **References** |
| --- | --- | --- | --- | --- | --- |
| 1 | ABCB1 | 5243 | P08183 | ATP-binding cassette, sub-family B (MDR/TAP), member 1 | Nafis, et al., 2015  [1] |
| 2 | AIFM1 | 9131 | O95831 | Apoptosis-inducing factor, mitochondrion-associated, 1 | Tanabe and Kanehisa, 2012 [2] |
| 3 | AKT1 | 207 | P31749 | V-akt murine thymoma viral oncogene homolog 1 | Tanabe and Kanehisa, 2012 [2] |
| 4 | AKT2 | 208 | P31751 | V-akt murine thymoma viral oncogene homolog 2 | Tanabe and Kanehisa, 2012 [2] |
| 5 | AKT3 | 10000 | Q9Y243 | V-akt murine thymoma viral oncogene homolog 3 | Tanabe and Kanehisa, 2012 [2] |
| 6 | APAF1 | 317 | O14727 | Apoptotic peptidase activating factor 1 | Holleman, et al., 2006; Tanabe and Kanehisa, 2012 [2, 3] |
| 7 | APC | 324 | P25054 | Adenomatous polyposis coli | Chu and Chen, 2008 [4] |
| 8 | APP | 351 | P05067 | Amyloid beta (A4) precursor protein | Chu and Chen, 2008 [4] |
| 9 | ATM | 472 | Q13315 | ATM serine/threonine kinase | Pal, et al., 2010; Tanabe and Kanehisa, 2012 [2, 5] |
| 10 | BAD | 572 | Q92934 | BCL2-associated agonist of cell death | Chu and Chen, 2008; Holleman, et al., 2006; Tanabe and Kanehisa, 2012 [2-4] |
| 11 | BAG1 | 573 | Q99933 | BCL2-associated athanogene | Chu and Chen, 2008; Holleman, et al., 2006 [3, 4] |
| 12 | BAG3 | 9531 | O95817 | BCL2-associated athanogene 3 | Chu and Chen, 2008; Holleman, et al., 2006 [3, 4] |
| 13 | BAG4 | 9530 | O95429 | BCL2-associated athanogene 4 | Chu and Chen, 2008; Holleman, et al., 2006 [3, 4] |
| 14 | BAG5 | 9529 | Q9UL15 | BCL2-associated athanogene 5 | Chu and Chen, 2008; Holleman, et al., 2006 [3, 4] |
| 15 | BAK1 | 578 | Q16611 | BCL2-antagonist/killer 1 | Chu and Chen, 2008; Holleman, et al., 2006 [3, 4] |
| 16 | BAX | 581 | Q07812 | BCL2-associated X protein | Chu and Chen, 2008; Holleman, et al., 2006; Tanabe and Kanehisa, 2012 [2-4] |
| 17 | BBC3 | 27113 | Q96PG8 | BCL2 binding component 3 | Holleman, et al., 2006 [3] |
| 18 | BCL2 | 596 | P10415 | B-cell CLL/lymphoma 2 | Chu and Chen, 2008; Holleman, et al., 2006; Pal, et al., 2010; Tanabe and Kanehisa, 2012 [2-5] |
| 19 | BCL2L1 | 598 | Q07817 | BCL2-like 1 | Chu and Chen, 2008; Holleman, et al., 2006; Tanabe and Kanehisa, 2012 [2-4] |
| 20 | BCL2L13 | 23786 | Q9BXK5 | BCL2-like 13 (apoptosis facilitator) | Holleman, et al., 2006 [3] |
| 21 | BCL6 | 604 | P41182 | B-cell CLL/lymphoma 6 | Chu and Chen, 2008 [4] |
| 22 | BFAR | 51283 | Q9NZS9 | Bifunctional apoptosis regulator | Holleman, et al., 2006 [3] |
| 23 | BID | 637 | P55957 | BH3 interacting domain death agonist | Chu and Chen, 2008; Holleman, et al., 2006; Tanabe and Kanehisa, 2012 [2-4] |
| 24 | BIK | 638 | Q13323 | BCL2-interacting killer (apoptosis-inducing) | Chu and Chen, 2008; Holleman, et al., 2006 [3, 4] |
| 25 | BIRC2 | 329 | Q13490 | Baculoviral IAP repeat containing 2 | Chu and Chen, 2008; Tanabe and Kanehisa, 2012 [2, 4] |
| 26 | BIRC3 | 330 | Q13489 | Baculoviral IAP repeat containing 3 | Chu and Chen, 2008; Tanabe and Kanehisa, 2012 [2, 4] |
| 27 | BIRC5 | 332 | O15392 | Baculoviral IAP repeat containing 5 | Chu and Chen, 2008 [4] |
| 28 | BIRC7 | 79444 | Q96CA5 | Baculoviral IAP repeat containing 7 | Holleman, et al., 2006 [3] |
| 29 | BNIP3L | 665 | O60238 | BCL2/adenovirus E1B 19kda interacting protein 3-like | Chu and Chen, 2008 [4] |
| 30 | BRCA1 | 672 | P38398 | Breast cancer 1, early onset | Pal, et al., 2010 [5] |
| 31 | BRCA2 | 675 | P51587 | Breast cancer 2, early onset | Pal, et al., 2010 [5] |
| 32 | CAPN1 | 823 | P07384 | Calpain 1, (mu/I) large subunit | Tanabe and Kanehisa, 2012 [2] |
| 33 | CAPN2 | 824 | P17655 | Calpain 2, (m/II) large subunit | Tanabe and Kanehisa, 2012 [2] |
| 34 | CARD8 | 22900 | Q9Y2G2 | Caspase recruitment domain family, member 8 | Holleman, et al., 2006 [3] |
| 35 | CASP1 | 834 | P29466 | Caspase 1, apoptosis-related cysteine peptidase | Chu and Chen, 2008; Holleman, et al., 2006 [3, 4] |
| 36 | CASP10 | 843 | Q92851 | Caspase 10, apoptosis-related cysteine peptidase | Chu and Chen, 2008; Holleman, et al., 2006 Tanabe and Kanehisa, 2012 [2-4] |
| 37 | CASP2 | 835 | P42575 | Caspase 2, apoptosis-related cysteine peptidase | Chu and Chen, 2008; Holleman, et al., 2006 [3, 4] |
| 38 | CASP3 | 836 | P42574 | Caspase 3, apoptosis-related cysteine peptidase | Chu and Chen, 2008; Holleman, et al., 2006; Tanabe and Kanehisa, 2012 [2-4] |
| 39 | CASP4 | 837 | P49662 | Caspase 4, apoptosis-related cysteine peptidase | Chu and Chen, 2008 [4] |
| 40 | CASP6 | 839 | P55212 | Caspase 6, apoptosis-related cysteine peptidase | Chu and Chen, 2008; Holleman, et al., 2006; Tanabe and Kanehisa, 2012 [2-4] |
| 41 | CASP7 | 840 | P55210 | Caspase 7, apoptosis-related cysteine peptidase | Chu and Chen, 2008; Holleman, et al., 2006; Tanabe and Kanehisa, 2012 [2-4] |
| 42 | CASP8 | 841 | Q14790 | Caspase 8, apoptosis-related cysteine peptidase | Chu and Chen, 2008; Holleman, et al., 2006; Pal, et al., 2010; Tanabe and Kanehisa, 2012 [2-5] |
| 43 | CASP8AP2 | 9994 | Q9UKL3 | Caspase 8 associated protein 2 | Holleman, et al., 2006 [3] |
| 44 | CASP9 | 842 | P55211 | Caspase 9, apoptosis-related cysteine peptidase | Holleman, et al., 2006; Tanabe and Kanehisa, 2012 [2, 3] |
| 45 | CCND1 | 595 | P24385 | Cyclin D1 | Chu and Chen, 2008 [4] |
| 46 | CDC6 | 990 | Q99741 | Cell division cycle 6 | Chu and Chen, 2008 [4] |
| 47 | CDKN1A | 1026 | P38936 | Cyclin-dependent kinase inhibitor 1A (p21, Cip1) | Chu and Chen, 2008 [4] |
| 48 | CDKN1B | 1027 | P46527 | Cyclin-dependent kinase inhibitor 1B (p27, Kip1) | Chu and Chen, 2008 [4] |
| 49 | CDKN2A | 1029 | P42771 | Cyclin-dependent kinase inhibitor 2A | Chu and Chen, 2008 [4] |
| 50 | CFLAR | 8837 | O15519 | CASP8 and FADD-like apoptosis regulator | Chu and Chen, 2008; Tanabe and Kanehisa, 2012 [2, 4] |
| 51 | CHEK2 | 11200 | O96017 | Checkpoint kinase 2 | Pal, et al., 2010 [5] |
| 52 | CHP1 | 11261 | Q99653 | Calcineurin-like EF-hand protein 1 | Tanabe and Kanehisa, 2012 [2] |
| 53 | CHP2 | 63928 | O43745 | Calcineurin-like EF-hand protein 2 | Tanabe and Kanehisa, 2012 [2] |
| 54 | CHUK | 1147 | O15111 | Conserved helix-loop-helix ubiquitous kinase | Tanabe and Kanehisa, 2012 [2] |
| 55 | CRADD | 8738 | P78560 | CASP2 and RIPK1 domain containing adaptor with death domain | Chu and Chen, 2008; Holleman, et al., 2006 [3, 4] |
| 56 | CSF2RB | 1439 | P32927 | Colony stimulating factor 2 receptor, beta, low-affinity (granulocyte-macrophage) | Tanabe and Kanehisa, 2012 [2] |
| 57 | CYCS | 54205 | P99999 | Cytochrome c, somatic | Chu and Chen, 2008; Holleman, et al., 2006; Tanabe and Kanehisa, 2012 [2-4] |
| 58 | DAPK1 | 1612 | P53355 | Death-associated protein kinase 1 | Holleman, et al., 2006 [3] |
| 59 | DAXX | 1616 | Q9UER7 | Death-domain associated protein | Holleman, et al., 2006 [3] |
| 60 | DCC | 1630 | P43146 | DCC netrin 1 receptor | Chu and Chen, 2008 [4] |
| 61 | DEDD | 9191 | O75618 | Death effector domain containing | Chu and Chen, 2008 [4] |
| 62 | DEDD2 | 162989 | Q8WXF8 | Death effector domain containing 2 | Chu and Chen, 2008 [4] |
| 63 | DFFA | 1676 | O00273 | DNA fragmentation factor, 45kda, alpha polypeptide | Holleman, et al., 2006; Tanabe and Kanehisa, 2012 [2, 3] |
| 64 | DFFB | 1677 | O76075 | DNA fragmentation factor, 40kda, beta polypeptide (caspase-activated dnase) | Holleman, et al., 2006; Tanabe and Kanehisa, 2012 [2, 3] |
| 65 | DIABLO | 56616 | Q9NR28 | Diablo, IAP-binding mitochondrial protein | Chu and Chen, 2008 [4] |
| 66 | E2F1 | 1869 | Q01094 | E2F transcription factor 1 | Chu and Chen, 2008 [4] |
| 67 | EGFR | 1956 | P00533 | Epidermal growth factor receptor | Chu and Chen, 2008 [4] |
| 68 | ENDOD1 | 23052 | O94919 | Endonuclease domain containing 1 | Tanabe and Kanehisa, 2012 [2] |
| 69 | ENDOG | 2021 | Q14249 | Endonuclease G | Tanabe and Kanehisa, 2012 [2] |
| 70 | EXOG | 9941 | Q9Y2C4 | Endo/exonuclease (5'-3'), endonuclease G-like | Tanabe and Kanehisa, 2012 [2] |
| 71 | FADD | 8772 | Q13158 | Fas (TNFRSF6)-associated via death domain | Holleman, et al., 2006; Tanabe and Kanehisa, 2012 [2, 3] |
| 72 | FAS | 355 | P25445 | Fas cell surface death receptor | Holleman, et al., 2006; Tanabe and Kanehisa, 2012 [2, 3] |
| 73 | FASLG | 356 | P48023 | Fas ligand (TNF superfamily, member 6) | Tanabe and Kanehisa, 2012 [2] |
| 74 | FGFR1 | 2260 | P11362 | Fibroblast growth factor receptor 1 | Chu and Chen, 2008 [4] |
| 75 | H2AFX | 3014 | P16104 | H2A histone family, member X | Pal, et al., 2010 [5] |
| 76 | HRAS | 3265 | P01112 | Harvey rat sarcoma viral oncogene homolog | Chu and Chen, 2008 [4] |
| 77 | HRK | 8739 | O00198 | Harakiri, BCL2 interacting protein | Chu and Chen, 2008; Holleman, et al., 2006 [3, 4] |
| 78 | HSPB1 | 3315 | P04792 | Heat shock 27kda protein 1 | Holleman, et al., 2006 [3] |
| 79 | HTRA2 | 27429 | O43464 | Htra serine peptidase 2 | Holleman, et al., 2006 [3] |
| 80 | IFNG | 3458 | P01579 | Interferon, gamma | Pal, et al., 2010 [5] |
| 81 | IGFBP3 | 3486 | P17936 | Insulin-like growth factor binding protein 3 | Chu and Chen, 2008 [4] |
| 82 | IKBKB | 3551 | O14920 | Inhibitor of kappa light polypeptide gene enhancer in B-cells, kinase beta | Tanabe and Kanehisa, 2012 [2] |
| 83 | IKBKG | 8517 | Q9Y6K9 | Inhibitor of kappa light polypeptide gene enhancer in B-cells, kinase gamma | Tanabe and Kanehisa, 2012 [2] |
| 84 | IL10 | 3586 | P22301 | Interleukin 10 | Pal, et al., 2011 [6] |
| 85 | IL1A | 3552 | P01583 | Interleukin 1, alpha | Tanabe and Kanehisa, 2012 [2] |
| 86 | IL1B | 3553 | P01584 | Interleukin 1, beta | Tanabe and Kanehisa, 2012 [2] |
| 87 | IL1R1 | 3554 | P14778 | Interleukin 1 receptor, type I | Tanabe and Kanehisa, 2012 [2] |
| 88 | IL1RAP | 3556 | Q9NPH3 | Interleukin 1 receptor accessory protein | Tanabe and Kanehisa, 2012 [2] |
| 89 | IL3 | 3562 | P08700 | Interleukin 3 | Tanabe and Kanehisa, 2012 [2] |
| 90 | IL3RA | 3563 | P26951 | Interleukin 3 receptor, alpha (low affinity) | Tanabe and Kanehisa, 2012 [2] |
| 91 | IL4 | 3565 | P05112 | Interleukin 4 | Nafis, et al., 2015 [1] |
| 92 | IL6 | 3569 | P05231 | Interleukin 6 | Pal, et al., 2011; Pal, et al., 2010 [5, 6] |
| 93 | IRAK1 | 3654 | P51617 | Interleukin-1 receptor-associated kinase 1 | Tanabe and Kanehisa, 2012 [2] |
| 94 | IRAK2 | 3656 | O43187 | Interleukin-1 receptor-associated kinase 2 | Tanabe and Kanehisa, 2012 [2] |
| 95 | IRAK3 | 11213 | Q9Y616 | Interleukin-1 receptor-associated kinase 3 | Tanabe and Kanehisa, 2012 [2] |
| 96 | IRAK4 | 51135 | Q9NWZ3 | Interleukin-1 receptor-associated kinase 4 | Tanabe and Kanehisa, 2012 [2] |
| 97 | KAT5 | 10524 | Q92993 | K(lysine) acetyltransferase 5 | Pal, et al., 2010 [5] |
| 98 | KRAS | 3845 | P01116 | Kirsten rat sarcoma viral oncogene homolog | Chu and Chen, 2008 [4] |
| 99 | MALT1 | 10892 | Q9UDY8 | Mucosa associated lymphoid tissue lymphoma translocation gene 1 | Chu and Chen, 2008 [4] |
| 100 | MAP3K14 | 9020 | Q99558 | Mitogen-activated protein kinase kinase kinase 14 | Tanabe and Kanehisa, 2012 [2] |
| 101 | MAP3K7 | 6885 | O43318 | Mitogen-activated protein kinase kinase kinase 7 | Chu and Chen, 2008  [4] |
| 102 | MAPK1 | 5594 | P28482 | Mitogen-activated protein kinase 1 | Chu and Chen, 2008  [4] |
| 103 | MAPK3 | 5595 | P27361 | Mitogen-activated protein kinase 3 | Chu and Chen, 2008  [4] |
| 104 | MCL1 | 4170 | Q07820 | Myeloid cell leukemia 1 | Chu and Chen, 2008; Holleman, et al., 2006 [3, 4] |
| 105 | MDM2 | 4193 | Q00987 | MDM2 proto-oncogene, E3 ubiquitin protein ligase | Gochhait, et al., 2009 [7] |
| 106 | MFN2 | 9927 | O95140 | Mitofusin 2 | Chu and Chen, 2008 [4] |
| 107 | MITF | 4286 | O75030 | Microphthalmia-associated transcription factor | Chu and Chen, 2008 [4] |
| 108 | MKI67 | 4288 | P46013 | Marker of proliferation Ki-67 | Chu and Chen, 2008 [4] |
| 109 | MMP9 | 4318 | P14780 | Matrix metallopeptidase 9 (gelatinase B, 92kda gelatinase, 92kda type IV collagenase) | Chu and Chen, 2008 [4] |
| 110 | MSH2 | 4436 | P43246 | Muts homolog 2 | Chu and Chen, 2008 [4] |
| 111 | MYC | 4609 | P01106 | V-myc avian myelocytomatosis viral oncogene homolog | Chu and Chen, 2008 [4] |
| 112 | MYD88 | 4615 | Q99836 | Myeloid differentiation primary response 88 | Tanabe and Kanehisa, 2012 [2] |
| 113 | NAIP | 4671 | Q13075 | NLR family, apoptosis inhibitory protein | Holleman, et al., 2006 [3] |
| 114 | NFKB1 | 4790 | P19838 | Nuclear factor of kappa light polypeptide gene enhancer in B-cells 1 | Chu and Chen, 2008; Tanabe and Kanehisa, 2012 [2, 4] |
| 115 | NFKBIA | 4792 | P25963 | Nuclear factor of kappa light polypeptide gene enhancer in B-cells inhibitor, alpha | Chu and Chen, 2008; Tanabe and Kanehisa, 2012 [2, 4] |
| 116 | NGF | 4803 | P01138 | Nerve growth factor (beta polypeptide) | Tanabe and Kanehisa, 2012 [2] |
| 117 | NLRC4 | 58484 | Q9NPP4 | NLR family, CARD domain containing 4 | Chu and Chen, 2008 [4] |
| 118 | NLRP1 | 22861 | Q9C000 | NLR family, pyrin domain containing 1 | Chu and Chen, 2008 [4] |
| 119 | NOD1 | 10392 | Q9Y239 | Nucleotide-binding oligomerization domain containing 1 | Chu and Chen, 2008 [4] |
| 120 | NRAS | 4893 | P01111 | Neuroblastoma RAS viral (v-ras) oncogene homolog | Chu and Chen, 2008 [4] |
| 121 | NTRK1 | 4914 | P04629 | Neurotrophic tyrosine kinase, receptor, type 1 | Tanabe and Kanehisa, 2012 [2] |
| 122 | PARP1 | 142 | P09874 | Poly (ADP-ribose) polymerase 1 | Holleman, et al., 2006 [3] |
| 123 | PCNA | 5111 | P12004 | Proliferating cell nuclear antigen | Chu and Chen, 2008 [4] |
| 124 | PEA15 | 8682 | Q15121 | Phosphoprotein enriched in astrocytes 15 | Chu and Chen, 2008 [4] |
| 125 | PECAM1 | 5175 | P16284 | Platelet/endothelial cell adhesion molecule 1 | Holleman, et al., 2006 [3] |
| 126 | PIK3CA | 5290 | P42336 | Phosphatidylinositol-4,5-bisphosphate 3-kinase, catalytic subunit alpha | Tanabe and Kanehisa, 2012 [2] |
| 127 | PIK3CB | 5291 | P42338 | Phosphatidylinositol-4,5-bisphosphate 3-kinase, catalytic subunit beta | Tanabe and Kanehisa, 2012 [2] |
| 128 | PIK3CD | 5293 | O00329 | Phosphatidylinositol-4,5-bisphosphate 3-kinase, catalytic subunit delta | Tanabe and Kanehisa, 2012 [2] |
| 129 | PIK3CG | 5294 | P48736 | Phosphatidylinositol-4,5-bisphosphate 3-kinase, catalytic subunit gamma | Tanabe and Kanehisa, 2012 [2] |
| 130 | PIK3R1 | 5295 | P27986 | Phosphoinositide-3-kinase, regulatory subunit 1 (alpha) | Tanabe and Kanehisa, 2012 [2] |
| 131 | PIK3R2 | 5296 | O00459 | Phosphoinositide-3-kinase, regulatory subunit 2 (beta) | Tanabe and Kanehisa, 2012 [2] |
| 132 | PIK3R3 | 8503 | Q92569 | Phosphoinositide-3-kinase, regulatory subunit 3 (gamma) | Tanabe and Kanehisa, 2012 [2] |
| 133 | PIK3R5 | 23533 | Q8WYR1 | Phosphoinositide-3-kinase, regulatory subunit 5 | Tanabe and Kanehisa, 2012 [2] |
| 134 | PKMYT1 | 9088 | Q99640 | Protein kinase, membrane associated tyrosine/threonine 1 | Chu and Chen, 2008 [4] |
| 135 | PML | 5371 | P29590 | Promyelocytic leukemia | Chu and Chen, 2008 [4] |
| 136 | PPP3CA | 5530 | Q08209 | Protein phosphatase 3, catalytic subunit, alpha isozyme | Tanabe and Kanehisa, 2012 [2] |
| 137 | PPP3CB | 5532 | P16298 | Protein phosphatase 3, catalytic subunit, beta isozyme | Tanabe and Kanehisa, 2012 [2] |
| 138 | PPP3CC | 5533 | P48454 | Protein phosphatase 3, catalytic subunit, gamma isozyme | Tanabe and Kanehisa, 2012 [2] |
| 139 | PPP3R1 | 5534 | P63098 | Protein phosphatase 3, regulatory subunit B, alpha | Tanabe and Kanehisa, 2012 [2] |
| 140 | PPP3R2 | 5535 | Q96LZ3 | Protein phosphatase 3, regulatory subunit B, beta | Tanabe and Kanehisa, 2012 [2] |
| 141 | PRKACA | 5566 | P17612 | Protein kinase, camp-dependent, catalytic, alpha | Tanabe and Kanehisa, 2012 [2] |
| 142 | PRKACB | 5567 | P22694 | Protein kinase, camp-dependent, catalytic, beta | Tanabe and Kanehisa, 2012 [2] |
| 143 | PRKACG | 5568 | P22612 | Protein kinase, camp-dependent, catalytic, gamma | Tanabe and Kanehisa, 2012 [2] |
| 144 | PRKAR1A | 5573 | P10644 | Protein kinase, camp-dependent, regulatory, type I, alpha | Tanabe and Kanehisa, 2012 [2] |
| 145 | PRKAR1B | 5575 | P31321 | Protein kinase, camp-dependent, regulatory, type I, beta | Tanabe and Kanehisa, 2012 [2] |
| 146 | PRKAR2A | 5576 | P13861 | Protein kinase, camp-dependent, regulatory, type II, alpha | Tanabe and Kanehisa, 2012 [2] |
| 147 | PRKAR2B | 5577 | P31323 | Protein kinase, camp-dependent, regulatory, type II, beta | Tanabe and Kanehisa, 2012 [2] |
| 148 | PRKX | 5613 | P51817 | Protein kinase, X-linked | Tanabe and Kanehisa, 2012 [2] |
| 149 | PSEN1 | 5663 | P49768 | Presenilin 1 | Chu and Chen, 2008 [4] |
| 150 | PTEN | 5728 | P60484 | Phosphatase and tensin homolog | Chu and Chen, 2008 [4] |
| 151 | RARG | 5916 | P13631 | Retinoic acid receptor, gamma | Chu and Chen, 2008 [4] |
| 152 | RELA | 5970 | Q04206 | V-rel avian reticuloendotheliosis viral oncogene homolog A | Tanabe and Kanehisa, 2012 [2] |
| 153 | RIPK1 | 8737 | Q13546 | Receptor (TNFRSF)-interacting serine-threonine kinase 1 | Holleman, et al., 2006; Tanabe and Kanehisa, 2012 [2, 3] |
| 154 | RNF8 | 9025 | O76064 | Ring finger protein 8, E3 ubiquitin protein ligase | Pal, et al., 2010 [5] |
| 155 | RPS27A | 6233 | P62979 | Ribosomal protein s27a | Nafis, et al., 2015 [1] |
| 156 | RTN4 | 57142 | Q9NQC3 | Reticulon 4 | Chu and Chen, 2008 [4] |
| 157 | SP1 | 6667 | P08047 | Sp1 transcription factor | Li, et al., 2014 [8] |
| 158 | TANK | 10010 | Q92844 | TRAF family member-associated NFKB activator | Holleman, et al., 2006 [3] |
| 159 | TGFB1 | 7040 | P01137 | Transforming growth factor, beta 1 | Chu and Chen, 2008; Pal, et al., 2010 [4, 5] |
| 160 | TNF | 7124 | P01375 | Tumor necrosis factor | Chu and Chen, 2008; Holleman, et al., 2006; Tanabe and Kanehisa, 2012 [2-4] |
| 161 | TNFRSF10A | 8797 | O00220 | Tumor necrosis factor receptor superfamily, member 10a | Tanabe and Kanehisa, 2012 [2] |
| 162 | TNFRSF10B | 8795 | O14763 | Tumor necrosis factor receptor superfamily, member 10b | Tanabe and Kanehisa, 2012 [2] |
| 163 | TNFRSF10C | 8794 | O14798 | Tumor necrosis factor receptor superfamily, member 10c, decoy without an intracellular domain | Tanabe and Kanehisa, 2012 [2] |
| 164 | TNFRSF10D | 8793 | Q9UBN6 | Tumor necrosis factor receptor superfamily, member 10d, decoy with truncated death domain | Tanabe and Kanehisa, 2012 [2] |
| 165 | TNFRSF12A | 51330 | Q9NP84 | Tumor necrosis factor receptor superfamily, member 12A | Holleman, et al., 2006 [3] |
| 166 | TNFRSF1A | 7132 | P19438 | Tumor necrosis factor receptor superfamily, member 1A | Holleman, et al., 2006 [3] |
| 167 | TNFRSF1B | 7133 | P20333 | Tumor necrosis factor receptor superfamily, member 1B | Holleman, et al., 2006 [3] |
| 168 | TNFRSF25 | 8718 | Q93038 | Tumor necrosis factor receptor superfamily, member 25 | Holleman, et al., 2006 [3] |
| 169 | TNFRSF6B | 8771 | O95407 | Tumor necrosis factor receptor superfamily, member 6b, decoy | Holleman, et al., 2006 [3] |
| 170 | TNFSF10 | 8743 | P50591 | Tumor necrosis factor (ligand) superfamily, member 10 | Holleman, et al., 2006; Pal, et al., 2010 [3, 5] |
| 171 | TP53 | 7157 | P04637 | Tumor protein p53 | Chu and Chen, 2008; Pal, et al., 2010; Tanabe and Kanehisa, 2012 [2, 4, 5] |
| 172 | TRADD | 8717 | Q15628 | TNFRSF1A-associated via death domain | Holleman, et al., 2006; Tanabe and Kanehisa, 2012 [2, 3] |
| 173 | TRAF1 | 7185 | Q13077 | TNF receptor-associated factor 1 | Chu and Chen, 2008; Holleman, et al., 2006  [3, 4] |
| 174 | TRAF2 | 7186 | Q12933 | TNF receptor-associated factor 2 | Holleman, et al., 2006; Tanabe and Kanehisa, 2012 [2, 3] |
| 175 | TRAF3 | 7187 | Q13114 | TNF receptor-associated factor 3 | Chu and Chen, 2008; Holleman, et al., 2006 [3, 4] |
| 176 | TRAF4 | 9618 | Q9BUZ4 | TNF receptor-associated factor 4 | Holleman, et al., 2006 [3] |
| 177 | TRAF6 | 7189 | Q9Y4K3 | TNF receptor-associated factor 6, E3 ubiquitin protein ligase | Chu and Chen, 2008; Holleman, et al., 2006 [3, 4] |
| 178 | UBA52 | 7311 | P62987 | Ubiquitin A-52 residue ribosomal protein fusion product 1 | Nafis, et al., 2015 [1] |
| 179 | UBB | 7314 | P0CG47 | Ubiquitin B | Nafis, et al., 2015 [1] |
| 180 | UBC | 7316 | P0CG48 | Ubiquitin C | Nafis, et al., 2015 [1] |
| 181 | VEGFA | 7422 | P15692 | Vascular endothelial growth factor A | Chu and Chen, 2008 [4] |
| 182 | XIAP | 331 | P98170 | X-linked inhibitor of apoptosis | Holleman, et al., 2006; Tanabe and Kanehisa, 2012 [2, 3] |

**References**

1. Nafis S, Kalaiarasan P, Brojen Singh RK, Husain M, Bamezai RN. Apoptosis regulatory protein-protein interaction demonstrates hierarchical scale-free fractal network. Brief Bioinform. 2015;16(4):675-99. Epub 2014/09/27. doi: 10.1093/bib/bbu036. PubMed PMID: 25256288.

2. Tanabe M, Kanehisa M. Using the KEGG database resource. Curr Protoc Bioinformatics. 2012;Chapter 1:Unit1 12. Epub 2012/06/16. doi: 10.1002/0471250953.bi0112s38. PubMed PMID: 22700311.

3. Holleman A, den Boer ML, de Menezes RX, Cheok MH, Cheng C, Kazemier KM, et al. The expression of 70 apoptosis genes in relation to lineage, genetic subtype, cellular drug resistance, and outcome in childhood acute lymphoblastic leukemia. Blood. 2006;107(2):769-76. Epub 2005/09/29. doi: 10.1182/blood-2005-07-2930. PubMed PMID: 16189266; PubMed Central PMCID: PMCPMC1895621.

4. Chu LH, Chen BS. Construction of a cancer-perturbed protein-protein interaction network for discovery of apoptosis drug targets. BMC Syst Biol. 2008;2:56. Epub 2008/07/02. doi: 10.1186/1752-0509-2-56. PubMed PMID: 18590547; PubMed Central PMCID: PMCPMC2488323.

5. Pal R, Srivastava N, Chopra R, Gochhait S, Gupta P, Prakash N, et al. Investigation of DNA damage response and apoptotic gene methylation pattern in sporadic breast tumors using high throughput quantitative DNA methylation analysis technology. Mol Cancer. 2010;9:303. Epub 2010/11/26. doi: 10.1186/1476-4598-9-303. PubMed PMID: 21092294; PubMed Central PMCID: PMCPMC3004830.

6. Pal R, Gochhait S, Chattopadhyay S, Gupta P, Prakash N, Agarwal G, et al. Functional implication of TRAIL -716 C/T promoter polymorphism on its in vitro and in vivo expression and the susceptibility to sporadic breast tumor. Breast Cancer Res Treat. 2011;126(2):333-43. Epub 2010/05/06. doi: 10.1007/s10549-010-0900-5. PubMed PMID: 20443055.

7. Gochhait S, Dar S, Pal R, Gupta P, Bamezai RN. Expression of DNA damage response genes indicate progressive breast tumors. Cancer Lett. 2009;273(2):305-11. Epub 2008/09/23. doi: 10.1016/j.canlet.2008.08.009. PubMed PMID: 18805634.

8. Li H, Zhang Y, Strose A, Tedesco D, Gurova K, Selivanova G. Integrated high-throughput analysis identifies Sp1 as a crucial determinant of p53-mediated apoptosis. Cell Death Differ. 2014;21(9):1493-502. Epub 2014/06/28. doi: 10.1038/cdd.2014.69. PubMed PMID: 24971482; PubMed Central PMCID: PMCPMC4131181.
